# DFCCNet: A Dense Flock of Chickens Counting Network Based on Density Map Regression

**DOI:** 10.3390/ani13233729

**Published:** 2023-12-01

**Authors:** Jinze Lv, Jinfeng Wang, Chaoda Peng, Qiong Huang

**Affiliations:** 1 College of Mathematics and Informatics, South China Agricultural University, Guangzhou 510642, China; 3170062@stu.scau.edu.cn (J.L.); chaodapeng@scau.edu.cn (C.P.);; 2 Key Laboratory of Smart Agriculture of Guangzhou, South China Agricultural University, Guangzhou 510642, China

**Keywords:** artificial intelligence, chicken counting, density map regression, feature fusion, multi-scaling

## Abstract

**Simple Summary:**

In large-scale chicken farming, monitoring flock density can help optimize feeding management and improve animal welfare. Traditional manual counting methods are not only prone to errors and omissions but also can cause harm to chickens. In this paper, a counting model for dense chicken flocks is proposed. The method can effectively estimate the number of chickens in real dense scenes.

**Abstract:**

With the development of artificial intelligence, automatically and accurately counting chickens has become a reality. However, insufficient lighting, irregular sizes, and dense flocks make this a challenging task. The existing methods cannot perform accurate and stable counting. In this article, a dense flock of chickens counting network (DFCCNet) is proposed based on density map regression, where features from different levels are merged using feature fusion to obtain more information for distinguishing chickens from the background, resulting in more stable counting results. Multi-scaling is used to detect and count chickens at various scales, which can improve the counting accuracy and ensure stable performance for chickens of different sizes. Feature convolution kernels are adopted to convolve feature maps, which can extract more accurate target information, reduce the impact of occlusion, and achieve more reliable and precise results. A dataset of dense flocks of chickens (namely Dense-Chicken) has been collected and constructed, which contains 600 images of 99,916 chickens, with labeled points and boxes. It can be accessed by researchers as benchmark data. The proposed method was compared with some state-of-the-art algorithms, to validate its effectiveness. With its robustness being verified by counting in three kinds of density situations, with the mean absolute error being 4.26, 9.85, and 19.17, respectively, and a speed of 16.15 FPS. DFCCNet provides an automatic and fast approach to counting chickens in a dense farming environment. It can be easily embedded into handheld devices for application in agricultural engineering.

## 1. Introduction

Chicken is one of the most popular meats in the world. Therefore, the chicken breeding industry has great economic value. In the process of chicken breeding, cageless rearing is more conducive to the growth of chickens than cage rearing [1,2]. Proper breeding density can improve the growth performance of chickens, as well as their immunity and carcass yield [3,4,5], while too intensive feeding can negatively affect the health of chickens [6,7]. Moreover, many steps such as welfare breeding [8], feed feeding, stocking, and slaughtering are needed to obtain accurate quantities. Therefore, rapid and accurate estimation of flock density is a very important research field.

Traditional poultry farming requires artificial observation of behavior and health status, manual feeding, and counting. This requires a lot of labor, which increases the cost of breeding. An irregular and inaccurate manual operation may cause harm to chickens. Moreover, due to the small space and high density of chickens, if the staff do not carry out complete disinfection before entering the chicken house, they may pass germs to the chickens, which spread quickly, causing serious losses [9]. It is obviously difficult for chicken farm managers to count manually, which can lead to wrong amounts, low efficiency, and subjective influences. In particular, overlapping chickens and different perspectives can cause large errors with complex backgrounds. These problems are common in large-scale breeding enterprises. Therefore, it is necessary to develop and implement efficient, automated, and highly accurate counting methods, so as to fully improve efficiency and truly realize intelligent and automated management.

Digital technology with intelligent monitoring methods is widely used in poultry health and welfare management [10], which can realize rapid, accurate, automatic, non-invasive monitoring in the process of poultry breeding, and help replace some labor-intensive tasks in poultry breeding. A novel fully automated and non-invasive monitoring system was proposed to detect lame behavior according to the important correlation between characteristic variables and broiler gait scores [11]. A real-time automatic feed weight monitoring system was developed to automatically detect the intake and weight of a single turkey, to study the feed conversion rate and feeding behavior of a single turkey in a population environment [12]. A segmentation algorithm could effectively separate a broiler from the background, extract the pose information of the broiler, and accurately and quickly identify the health status of the broiler with an accuracy rate of 99.47% [13]. However, these methods belong to shallow learning, which limits performance when dealing with complex problems, due to a lack of deep topology and big data support.

Deep learning is a method of data representation-based learning that consists of multiple processing layers, to learn data representations with multiple levels of abstraction [14]. An attention encoder and convolutional neural network (CNN) were used to detect and classify chicks in different living stages, with an accuracy of 95.2% [15]. CNN was used to monitor the opening beak behavior, spatial dispersion, and movement of chickens [16]. A deep neural network (DNN) and cross-entropy in information theory were used to train rooster and hen classifiers with an average accuracy of 96.85%, which provided a feasible method for the estimation of sex ratio [17]. A camera-based system was developed to monitor chickens and detect injuries caused by pecking at each other using neural networks [18]. A DNN-based pose estimation method was first applied to classify and identify the poses of broilers [19]. Experiments showed that chickens in standing, walking, running, eating, and resting states could be identified.

Counting refers to estimating the number of objects in a target area, to obtain information and perform timely control operations, and is currently widely used in various fields [20,21,22]. In the field of chicken counting, some researchers have conducted related studies. One study applied a localized fully convolutional network (LCFCN) to count and locate chickens from images of the pins with an average absolute error (MAE) of 0.56 [23]. A fully convolutional network termed DenseFCN was designed, and a point-supervised method was used to count chickens in the image, with an accuracy of 93.84% and speed and 9.27 frames per second (FPS) [24]. A novel framework called YOLOX-birth growth death (Y-BGD) was proposed for automatic and accurate cage-free broiler counting, with an average accuracy of 98.13% [25]. A automatic system based on YOLOv5 was applied to count chickens in the image with an average accuracy rate of 95.87% [26]. We analyzed the datasets used in these studies, in which [23,24,25] counted chickens in sparse scenes, with an average number of chickens per image of 27.89, 24.36, and 2.38, respectively. Ref. [26] counted chickens in both sparse and dense scenes, with an average number of chickens per picture of 155.37. However, the counting objects in all of these studies were large chickens. In real farming environments, large and small chickens are not the same in farming scenes; the same number of small chickens are farmed in smaller areas, and small chickens are more likely to congregate together, thus creating more serious occlusion and shadowing. More research is needed to address this situation.

At present, the mainstream counting technologies can be divided into two types, namely object detection and density map regression. Object detection can identify objects in images and determine their size and position using prediction boxes. The number of prediction boxes can be used to count. Object detection is mainly applied to two counting scenes: one is to directly detect and count chickens in sparse scenes [26], and the other is to count chickens passing through the counting line in the corridor by combining tracking algorithms [25]. But the accuracy is greatly affected in dense environments with heavy occlusion. Density map regression is mainly used in high-density scenes, which can obtain accurate counting by learning the mapping relationship between the picture and the real density map. This method has been applied in aquaculture. A lightweight fish counting model based on density plot regression was used for counting high-density fish [27]. Multi-scale modules and attention mechanisms were integrated into a network based on density map regression in fish counting, with an accuracy of 97.1% [28]. Although these experiments were conducted in a dense environment, their experimental environment was also ideal. Serious occlusion and complex backgrounds could still lead to poor stability and accuracy of the model, and it is necessary to further improve the performance.

Lightweight models are crucial in practical applications. Deep learning models usually require a large amount of computational resources, such as CPU, GPU, and so on. If the computational burden is too heavy, this will cause the model to run slowly, or even not be able to run on resource-constrained devices. This will make the model unable to meet the requirements of real-time calculation and portability, hindering its application in practice. Therefore, it is important to take into account the weight of the model, while ensuring the correctness of the model.

Aiming at avoiding heavy computational effort, and further improving the chicken counting accuracy under serious occlusion and complex environments, a lightweight model based on density map regression is proposed. The main contributions can be summarized as follows:

(1) A lightweight framework, DFCCNet, was designed by improving a feature convolution kernels module and proposing a density map module, satisfying the requirements for fast computing power and high detection accuracy in dense-flock chicken-counting tasks;
(2) Feature fusion is adopted to obtain more feature information with insufficient lighting; a multi-scaling mechanism can be used for solving irregular sizes; and feature convolution kernels are employed to address serious occlusion;
(3) A self-built dataset called Dense-Chicken was collected and constructed in a complex feeding environment, which contains 600 images of 99,916 chickens, in dot annotation files and box annotation files. It can be shared for researchers.

The rest of this article is organized as follows: Section 2 provides an overall description of the proposed models and methods. Section 3 describes the details of dataset preparation, which was used to train and evaluate the proposed network. The experimental results and a performance analysis of the proposed network are presented. Finally, Section 4 concludes the work by providing a summary and prospects of the proposed technique.

## 2. Materials and Methods

In this section, the overall framework of DFCCNet is first introduced, and then the feature convolution kernel generation module and counting module are explained. Finally, the loss function is demonstrated. The code is available at https://github.com/2226450890/DFCCNet (accessed on 22 November 2023).

### 2.1. Overall Framework

**Figure 1:**
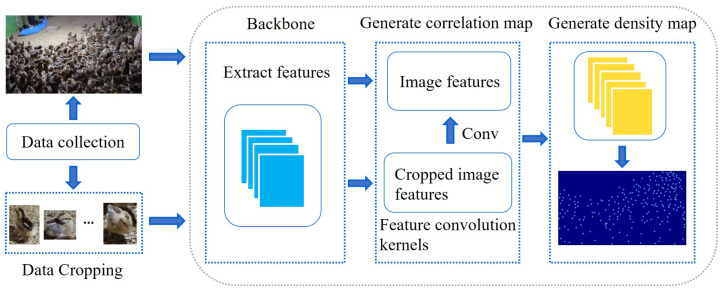
The overall framework of DFCCNet.

The overall framework of DFCCNet is shown in Figure 1. First, the original image and the cropped image are fed into the backbone module for feature extraction, to obtain image features and cropped image features, respectively. Then, in the correlation map generation module, the feature map of the cropped image is used as a feature convolution kernel to convolve the image features, to generate the correlation map. Finally, in the density map generation module, the resolution of the correlation map is restored to the original image size and the density map is generated. In particular, the parameters of the feature convolution kernel remain constant throughout training and testing, and it only needs to be generated once before the counting model is trained. The model makes direct calls to the feature convolution kernel during training and testing.Therefore, depending on whether the model parameters are changed and the order of execution, DFCCNet is divided into two parts: the feature convolution kernel generation module, and the counting module.

### 2.2. Feature Convolution Kernel Generation Module

**Figure 2:**
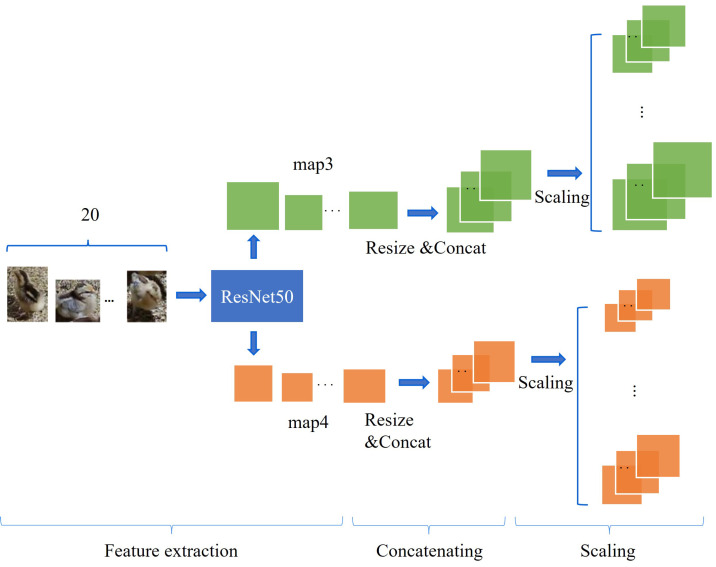
The structure of the feature convolution kernel generation module. The whole process is divided into three stages: feature extraction, concatenating, and scaling. Where the green and orange blocks represent the output of the third and fourth residual blocks, respectively.

In this study, the images contain a lot of useless background information that can adversely affect the counting of chickens. In order to count objects from different categories, Ref. [29] proposed an adaptive method. The input information for this method consists of the image and the box information of the counting target. Before generating the density map, image features located within these boxes are used as convolution kernels to perform convolution operations with the overall image features, to generate a correlation map focused on the counting objects. Inspired by this approach, feature convolution kernels are generated for enhancing the correlation between counting objects and feature maps, while filtering useless background information. Unlike the original method, in this study, since the counting objects are chickens of the same breed and age, and the morphological differences between different chickens in the images are small, generating different convolution kernels to improve the correlation of the feature maps during training was unnecessary. In this paper, the feature convolution kernel generation module is independent as the pre-module for counting, and the structure of this module is shown in Figure 2, which first adopts multiple images of a single chicken for feature extraction, and then the feature maps of different images are resized to the same size and fused to form a feature convolution kernel. During the whole model training and testing period, the feature convolution kernel only needs to be generated once. Although this can reduce the computational consumption, the quality of the feature convolution kernel needs to be considered. ResNet [30] has a wide range of applications in agriculture due to its excellent model performance [31,32,33]. ImageNet is a large-scale dataset, and by pretraining on this dataset, a model can be given good feature extraction capability [34]. Therefore, the pretrained ResNet in the feature convolutional kernel generation module is used for feature extraction, to ensure the quality of the convolutional kernel. Various versions of ResNet were used for comparative experiments, and finally ResNet50 was selected.

Although the experimental subjects were all chickens of the same breed and age, they presented different sizes in the images. Single-scale feature extraction can lead to the loss of multi-scale information. In order to solve this problem, multi-scale scaling methods are widely adopted [35,36]. In this paper, multi-scale scaling is applied to the feature convolution kernel, which ensures that the subsequently generated correlation maps can focus on the multi-scale chicken information, and thus accurately count chickens of different sizes in the images.

### 2.3. Counting Module

**Figure 3:**
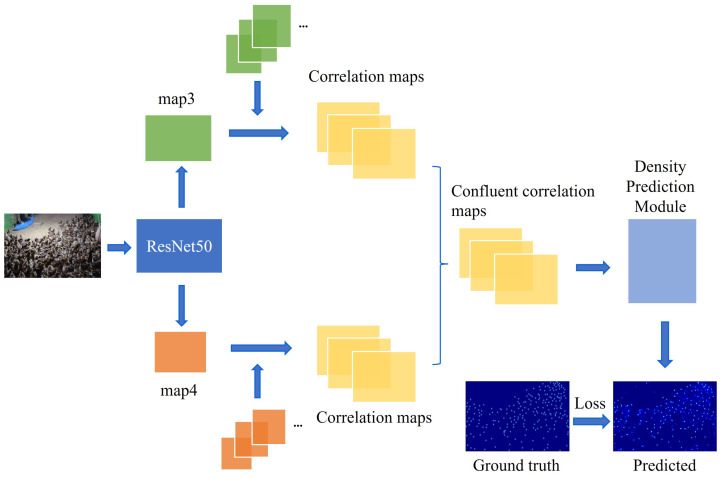
The structure of counting module.

The framework of the counting module is presented in Figure 3. First, the image is input to ResNet50 to extract features, and feature convolution kernels are used to generate the correlation maps. Then, the correlation maps are adjusted to the same size and concatenated together for input into the density map prediction module. Finally, the predicted density map is generated. During the training phase, the loss between the predicted density map and the ground truth is calculated to update the model parameters. During the testing phase, the sum of the pixel values of the predicted density map is used as the counting result. In the counting module, two strategies are used to improve the counting performance.

(1) Transfer learning. In this research, data annotation became a time-consuming and cumbersome task, due to the high density and complex environment. Although a significant amount of time and effort was spent on data annotation, limited data still affected the performance of the model. Transfer learning provided us with new possibilities and can effectively apply knowledge learned in one task or field to other related tasks or fields, thereby greatly improving the learning ability and generalization of the model [37]. Through transfer learning, the trained models could be used as a base to quickly retrain new tasks, without the need to train the entire network from scratch. This method not only saved a lot of time and computational resources, but also improved the performance of the model under limited data conditions [38,39]. As with the feature convolution kernel generation module, the pretrained ResNet50 is used for feature extraction.

(2) Feature fusion. The feature maps output from different depths of the convolutional layers have different information: shallow feature maps are rich in spatial information, and deep feature maps are rich in semantic information. Fusion of feature maps from different layers can improve network representation and enhance information acquisition, effectively improving model performance [40,41]. In this paper, the feature maps and feature convolution kernels output from the third residual block and the fourth residual block of ResNet50 are utilized to generate the correlation maps of different layers, which are fused to obtain the final correlation map. This correlation map can be used to generate a high-quality density map for counting.

**Figure 4:**
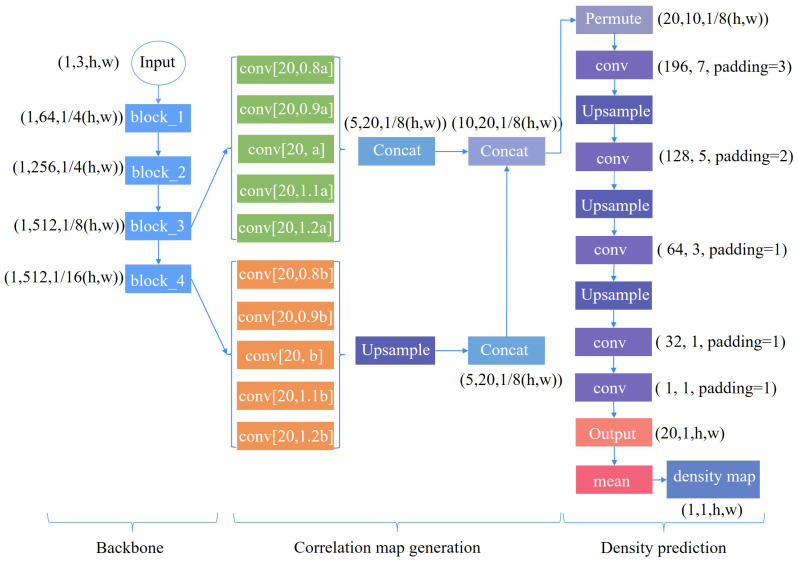
Details of the counting module. In this figure, *h* and *w* refer to the height and width of the input image, and *a* and *b* refer to the characteristic convolution kernel sizes of the output of the third and fourth blocks. Since the feature convolution kernel generated earlier uses 20 cropped images, the dimension of the convolution kernel is 20 in the correlation map generation module. The scale_factor for all upsampling operations was set to 2.

The details the of counting module are shown in Figure 4, which is divided into three modules; i.e., feature extraction, correlation map generation, and density map prediction. The feature convolution kernels and feature map obtained by the third block are used to obtain a series of correlation maps. The same operation is performed on the fourth block. In order to finally concatenate the correlation maps, the correlation maps output by the fourth block need to be upsampled. In the density map prediction module, five convolution kernels and three upsampling operations are used, where the first four convolution kernels are designed to restore the output size to the input size and the last convolution kernel is designed to predict the density map. Finally, the sum of the pixel values of the density map is used as the counting result.

### 2.4. Loss Function

In this paper, mean squared error (MSE) is used as the main body of the loss function, which means the average of the squared distance between the predicted value and the true value. MSE is defined in Equation (1).

(1) $$MSE=\frac{1}{N}\sum_{i=1}^{N}{\left(Z_{i}-{\hat{Z}}_{i}\right)}^{2}$$

where *N* represents the number of images, $Z_{i}$ represents the real density map of the *i*-th image, and ${\hat{Z}}_{i}$ represents the predicted density map. However, MSE is sensitive to outliers and susceptible to noise (including different sizes, occlusion, etc.) in the dense-counting field, which leads to inaccurate prediction in high-density areas. Thus, we added a constraint to the MSE that uses the random occlusion target for additional MSE to reduce noise interference. The random occlusion target is labeled on each image in the training set. The loss function is defined in Equation (2).

(2) Loss=MSE+λMSE(occlusion_target)

The former MSE calculates the error between the predicted density map and the true density map. The latter part is the error between the predicted density map corresponding to the random occlusion target and its real density map. λ is a hyperparameter set as $1\times 10^{-9}$.

## 3. Experiments and Discussion

In this section, a series of experiments are reported. (1) Each strategy was incrementally added to the counting network to determine its effectiveness, and the most appropriate parameter settings were identified. (2) The validation and testing sets were divided into different densities to verify the robustness of the model. (3) Comparison experiments between DFCCNet and some state-of-the-art methods were executed, to show the counting performance of DFCCNet.

### 3.1. Dataset

**Figure 5:**
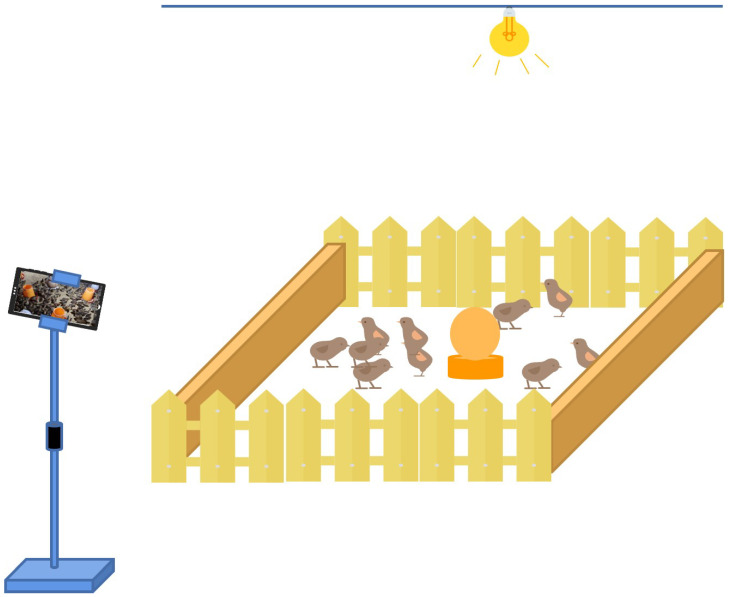
Experimental data acquisition device.

The experimental data were collected from a chicken farm in Guangdong Province in China in October 2021. The collected subjects were week-old chickens. The equipment for data acquisition is shown in Figure 5. The height of the chicken farm fence was 50 cm. During the data collecting process, our devices mainly included a mobile phone with the function of recording video and a bracket. The bracket was used to fix the mobile phone during the video process, and the length of the bracket could be adjusted in the range of 50–100 cm. Then, diverse data could be obtained from different angles in different breeding areas, which enhanced the generalization ability of the model. Finally, we obtained 20 videos with a resolution of 1280 × 720 pixels and a duration of 3 min, which had a frame rate of 30 frames per second.

#### 3.1.1. Dataset Construction

A total of 1800 images were intercepted in videos by cutting 1 frame every 60 frames. All images were then examined and images with motion blur and a similar chicken distribution were removed, retaining a total of 600 images. The data were divided into a training set, validation set, and testing set, in a ratio of 7:1:2. And the images in the different sets were from different videos. The distribution of the dataset is shown in Table 1. In the data labeling stage, two image annotation tools, i.e., Labelme and LabelImg, were adopted to obtain JSON files containing point coordinate information and XML files containing box coordinate information. The representative dataset is available at https://stuscaueducn-my.sharepoint.com/:u:/g/personal/3170062_stu_scau_edu_cn/ETT-vDigmvZBu6EgSRtSn0sBnNHLojY_tDmiVaoZteVP3g?e=rGa2yO (accessed on 15 December 2022).

**Figure 6:**
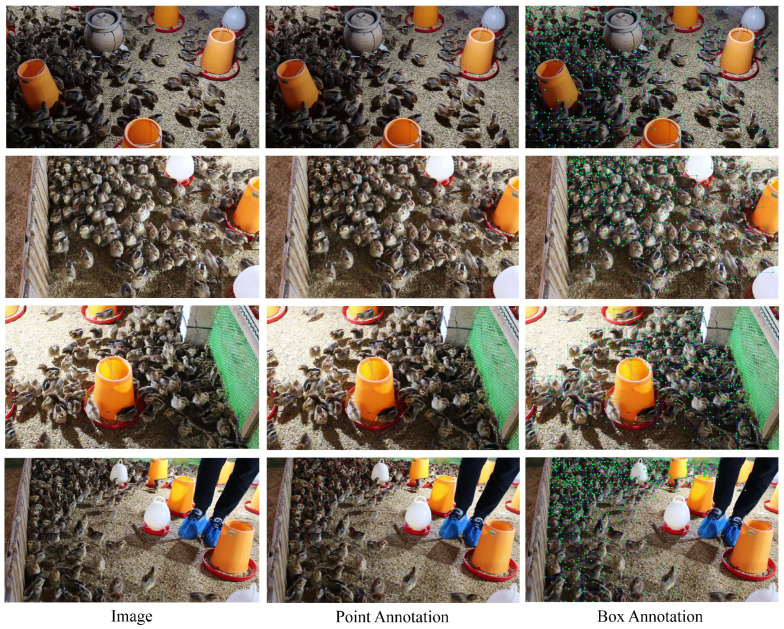
Images and annotation files in different scenes.

Some scenes in the dataset are shown in Figure 6. The challenges of counting a dense flock of chickens are presented as follows:

(1) Complex background in the pictures: Chickens in different areas are exposed to different intensities of light, in which it is difficult to distinguish chickens from the background;
(2) Occlusion situation: Since some feeders are placed in the breeding area, these devices can block the chickens to some extent. On the other hand, the high density of chickens and their habit of gathering also cause occlusion;
(3) Pixel blur: Chickens like to move and sometimes move quickly, which can cause pixel blur;
(4) Different scales: Since the object has the characteristics of being large in proximity and small in the distance, there will be many chickens of different sizes in the image. This requires a model to have strong feature extraction capabilities;
(5) Different numbers: The density and number of chickens in different regions are different, which will challenge the generalization of a model.

#### 3.1.2. Data Preprocessing

The spatial distribution information of the chickens needed to be generated according to the ground truth before training. Gaussian smoothing with an adaptive size window was used to generate a density map. First, a point annotation map was given, in which each point was located in the approximate center of the object. Second, the distances between each point and its nearest neighbor were calculated and averaged. Finally, this average distance was used as the size of the Gaussian window to generate the target density map.

In the model, feature convolution kernels fusing the features of single chickens were adopted, to generate correlation maps with better correlation with chickens.

**Figure 7:**
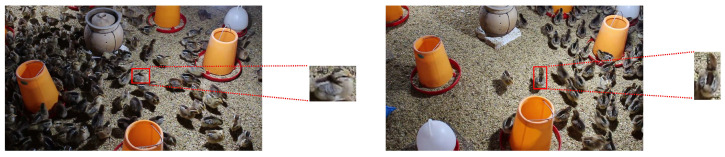
Image cropping. The chickens cut out are intact, with different sizes and directions.

Therefore, single chickens with different sizes and orientations were obtained by cropping images. The process of cropping image is shown in Figure 7.

### 3.2. Experimental Environmental and Metrics

The experimental environment of DFCCNet is listed in Table 2. The PyTorch framework for deep learning was adopted.

In this paper, mean absolute error (MAE), root mean squared error (RMSE), and mean normalized absolute error (NAE) were used as the main evaluation metrics in the counting experiments [42,43,44]. MAE is the average error between the predicted values and ground truth in counting, which was used to evaluate the accuracy of the model. RMSE is the dispersion of the error between the predicted values and ground truth, reflecting the stability of the model. NAE is the normalized MAE. They are defined in Equations (3), (4) and (5), respectively.

(3) $$MAE=\frac{1}{N}\sum_{i=1}^{N}\left|y_{i}-{\hat{y}}_{i}\right|$$

(4) $$RMSE=\sqrt{\frac{1}{N}\sum_{i=1}^{N}{\left(y_{i}-{\hat{y}}_{i}\right)}^{2}}$$

(5) $$NAE=\frac{1}{N}\sum_{i=1}^{N}\frac{\left|y_{i}-{\hat{y}}_{i}\right|}{y_{i}}$$

where *N* is the number of images, $y_{i}$ represents the real number of chickens in the *i*-th image, and ${\hat{y}}_{i}$ represents the predicted number of chickens in the *i*-th image. The smaller MAE and RMSE, the higher the accuracy and stability of the model.

In addition, the parameter amount and frames per second (FPS) were used for evaluating the performance of the model. Parameter amount determines the size of the model. If a model is for use on a mobile platform, fewer parameters should be set to meet lightweight requirements. FPS was used to test the efficiency and speed of the model.

### 3.3. Model Validation with Different Strategies

In this section, transfer learning, feature convolution kernel, feature fusion, and improved loss function were sequentially added to the network for counting.

#### 3.3.1. Transfer Learning

In this paper, a pretrained ResNet50 network was used to extract image features.ResNet is a deep learning network with many different versions, such as ResNet18, ResNet34, ResNet50, and ResNet101, which have different depths and sizes. The performance of a model improves as the depth of the network increases, but the model becomes more complex and has a larger size. In order to select the most appropriate version of ResNet and to verify the effectiveness of transfer learning, different models were used to extract image features. The experimental results are shown in Table 3, where the MAE of the models were all >20 without using transfer learning, while the MAE decreased dramatically when transfer learning was used. In addition, the MAE of ResNet50 and ResNet101 were significantly smaller than those of ResNet18 and ResNet34 when transfer learning was used. Although the error of ResNet101 was slightly smaller than ResNet50, its size was much larger. Therefore, considering the performance and size of the models, the pretrained ResNet50 was used to extract image features.

#### 3.3.2. Feature Convolution Kernel

In order to improve the relevance of the image features to the chickens, and to reduce the interference of the complex background, the ordinary convolution kernel was replaced with a feature convolution kernel. During the feature convolution generation process, features from multiple single chicken images were fused and multi-scale scaling was performed. The following experiments explored these factors:

(1) Feature Fusion. Although the counting objects in this paper were chickens of the same breed and age, the feature map of one image did not provide an accurate representation of all chickens, due to their different postures. A feature convolution kernel that fuses enough features is beneficial for improving the relevance of the image features to chickens during the counting process. To find the most suitable number of features, a series of experiments were executed. The experimental results are shown in Table 4. The results show that the performance of the model improved as the number of features increased. However, the model performance no longer improved after a certain number was reached. This is because extracting a large number of image features is conducive to enriching the feature information of the chickens, but excessive information will lead to redundant feature information. Finally, the features of twenty single chicken images were extracted for the subsequent experiments.

(2) Features Multi-scaling. A multi-scaling strategy was adopted to obtain rich target features by adding convolution kernels with different scales. The length and width of most chickens in the image were between 50 and 150 pixels. In the stage of feature convolution kernel generation, the feature maps could represent the features of single chickens with 100×100 pixels. Therefore, the scales were set from 0.5 to 1.5, to allow the model to accurately count chickens with different scales. A series of experiments were implemented to find the most suitable strategy. The results are shown in Table 5. The magnification and reduction factors were set consistently. New scales were added to the combination of scales with the best result. This is conducive to counting chickens of different scales, through adding feature convolution kernels with multiple scales. But too much accumulation can produce redundant information, such that the performance cannot be improved. Finally, the best result was obtained by setting multiple scales of 0.8, 0.9, 1.0, 1.1, and 1.2.

#### 3.3.3. Feature Fusion

In addition to the feature fusion used due to the introduction of feature convolution kernels, feature fusion was used to concatenate features from the different layers during the correlation map generation stage. Extracting features using the fifth residual block of ResNet50 resulted in a significant increase in the number of model parameters; therefore, in order to reduce the computational consumption, the outputs of the third and fourth residual blocks were used for the experiments. The experimental results are shown in Table 6. It is obvious that concatenating the correlation maps of the third block and the fourth block obtained the best result. The richer the feature information obtained, the higher the quality of the density map generated using the fusion strategy.

#### 3.3.4. Improved Loss Function

The loss function in this article used additional MSE. Different values of the hyperparameter (λ) were set for the experiments. The experimental results are shown in Table 7, the best results were obtained when the value of λ was set to $1\times 10^{-9}$. Compared to the original MSE, the addition of the extra MSE reduced the MAE by 0.29 for the validation set and 0.32 for the test set.

**Figure 8:**
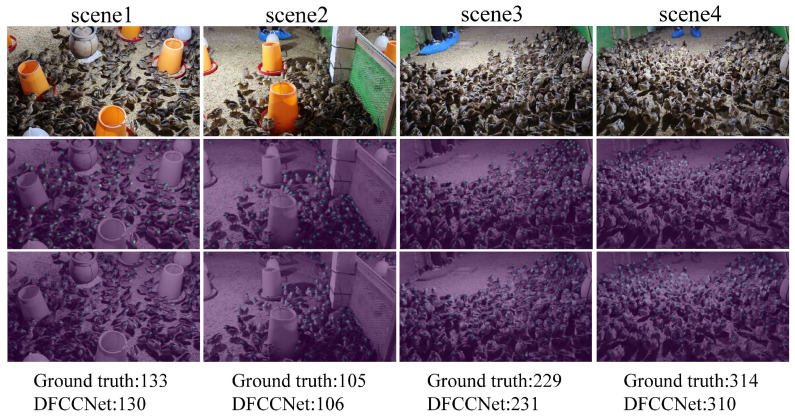
Visualization of counting results. The first line shows the original image, the second line shows the ground truth, and the last line shows the visualization output of DFCCNet. For better observation, the gray scale map was combined with the density map.

In the series of experiments above, various strategies were sequentially added to the network to perform the counting task, and the experiments verified their effectiveness. DFCCNet adopted all of these strategies and achieved accurate counting results, as shown in Figure 8.

### 3.4. Robustness Testing

**Figure 9:**
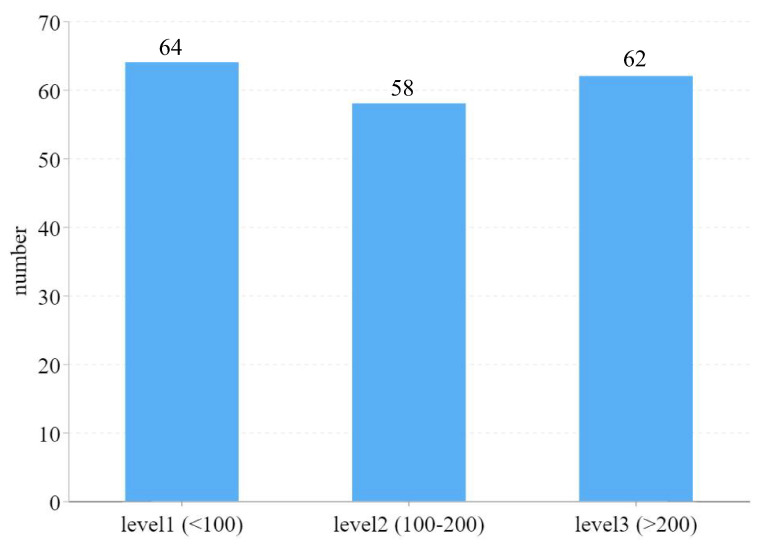
Distribution of data for different density levels.

In order to verify the robustness of DFCCNet, the validation set and testing set were divided into different groups with different density levels, according to the number of targets. The detailed distribution of the data is shown in Figure 9. The data with three density levels were used as validation sets, to verify the robustness of the model. CSRNet [45] was compared as a baseline. The results are presented in Table 8. DFCCNet achieved smaller counting errors than CSRNet at the same level, and the NAE was less than 0.1 for all levels. Therefore, DFCCNet could maintain stable performance at different densities.

### 3.5. Comparison with Other Methods

To further analyze the performance of DFCCNet, six state-of-the-art methods, including YOLOv5x [46], YOLOv7x [47], multi-column convolutional neural network (MCNN) [48], CSRNet, context aware network (CAN) [49], and segmentation guided attention networks (SGANet) [50] were used to compare with DFCCNet on the same training set, validation set, and testing set. The experimental results are shown in Table 9.

**Figure 10:**
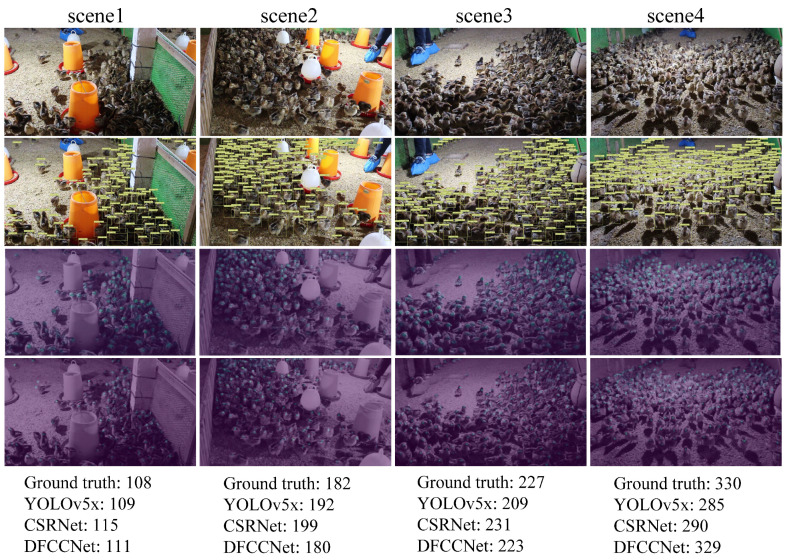
Comparison of yolov5x, CSRNet, and DFCCNet. For the same image, the result of DFCCNet was closer to the ground truth.

First, DFCCNet was compared with two advanced object detection algorithms, YOLOv5x and YOLOv7x. However, it was difficult for these two methods to detect accurately individual objects in dense groups, due to occlusion phenomena. It is obvious that they had large MAE values in the experiment. Compared to YOLOv5x, the MAE of DFCCNet was reduced by 2.66 for the validation set and 3.41 for the test set. Although DFCCNet’s FPS decreased by 20.55 compared to YOLOv5x, its number of parameters decreased by 69.31 MB. For the other density map regression methods, it can be seen that the parameters of MCNN were only 0.13 MB, and FPS reached 56.68. However, the three-column convolution kernels used by MCNN were too large in size. It could not extract features well for some small targets, resulting in a low accuracy. CSRNet, CANNet, and SGANet had a higher accuracy. But they are more complex, making them slow, and had a FPS of less than 10. Compared to CSRNet, DFCCNet showed a significant improvement in performance. The MAE was reduced by 1.12 on the validation set and 2.13 on the test set, and the FPS was improved by 7.72, while the parameters were increased by only 1.63 MB. Counting tasks in real scenery need to be implemented on mobile devices, which requires important considerations such as accuracy, size, and speed. In comparison, DFCCNet had the best accuracy, while maintaining acceptable parameters with 17.89 MB and FPS with 16.15, which can better meet the requirements of mobile devices. As shown in Figure 10, some renderings of DFCCNet, YOLOv5x, and CSRNet are presented. The first row shows some images were counted, and the last three rows list the counting results of YOLOv5x, CSRNet, and DFCCNet, respectively. It can be seen that YOLOv5x could obtain good counting result when the occlusion was not serious in scene1, but accurate counting was hindered in the case of serious occlusion in scene3 and scene4. In sparse scenes, density map regression, such as in DFCCNet and CSRNet, was not superior to object detection method, i.e., YOLOv5x. But they could achieve a higher counting accuracy than YOLOv5x in dense scenes. It is obvious that the counting results of DFCCNet were better than those of the classical model, i.e., CSRNet.

**Figure 11:**
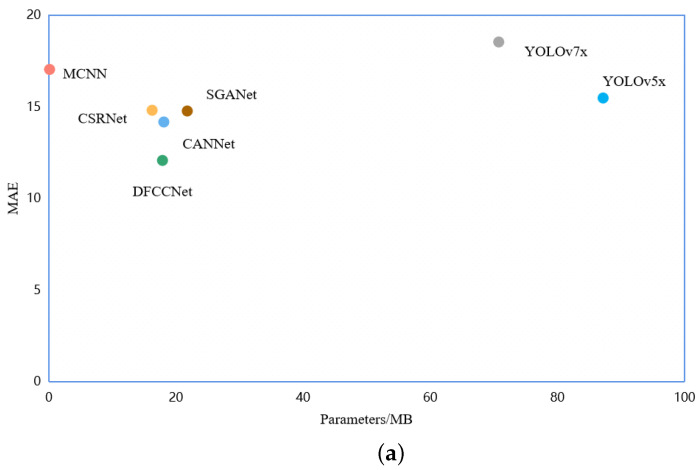

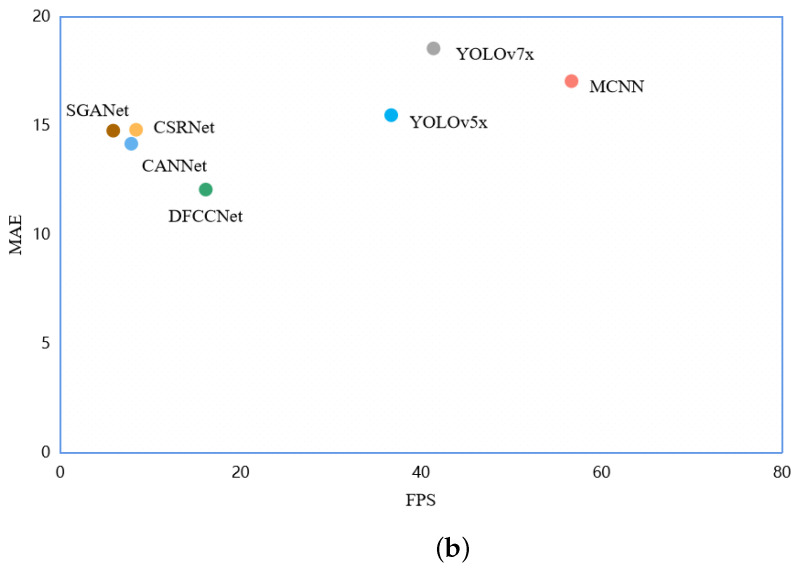
Comparing the test MAE, parameters, and FPS of the different methods. (**a**) The relationship between MAE and parameters. (**b**) The relationship between MAE and FPS.

In addition, the values of MAE with the parameters and FPS respectively for the different models are shown in Figure 11. The methods with a density map have fewer parameters and smaller errors, while the object detection methods are faster. It can be seen that DCCNet is more suitable for the counting task for dense flocks of chickens.

### 3.6. Mobile Application

**Figure 12:**
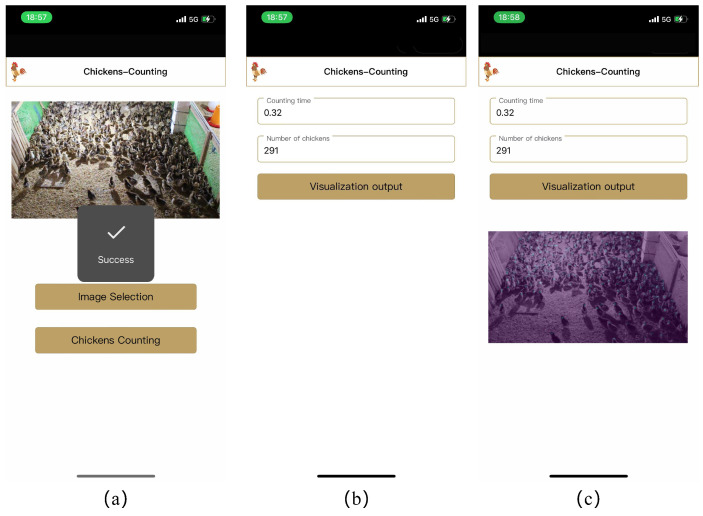
Chickens counting mobile app user interface. (**a**) Picture acquisition screen, (**b**) counting result, (**c**) visualization result.

To enable DFCCNet to run on mobile devices and make the system easily accessible to end users, a mobile application (named Chickens-Counting) was developed. During the deployment of the model, the format of the model was transformed into Core ML (IOS), a format that can be used by mobile frameworks. In order to improve the performance of the model and reduce memory usage, quantization methods were used to compress the trained model. Sample screens of the app are shown in Figure 12. The app can ask the camera to take a picture or select a picture from the album, then the picture is passed to the API of the counting module. The API returns the time consumed by the counting process and the result of counting, and a visual image of the counting result is displayed in the screen. The app was tested on an iPhone 12 and the time required to count a single picture was 0.3 ± 0.05 s, which meets the counting requirements for mobile devices.

## 4. Conclusions

In modern farms, fast and accurate automatic counting is essential for chicken farming. In this paper, a dense-flock chicken-counting network termed DFCCNet was proposed based on density map regression, which implements accurate counting through generating high-quality density maps. In addition, a dataset of dense flocks of chickens in complex environments, called Dense-Chicken, was collected and constructed for training and evaluation. The experimental results showed that the density map regression method was superior to the object detection method in dense situations. Compared with some advanced density map regression methods, DFCCNet achieved good results in dense and complex scenes, with a small number of parameters and fast counting speed. As a result, DFCCNet can be embedded and deployed on mobile devices used for chicken farming. It can also provide scientific and theoretical guidance for fast and accurate counting of dense flocks of chickens.

## Figures and Tables

**Table 1:** The amounts and distribution of the proposed dataset.

Datasets	Images	Chickens	Average
Complete dataset	600	99,916	166
Training Set	416	70,777	170
Validation Set	61	9657	158
Testing Set	123	19,482	158

**Table 2:** Experimental environment.

Configuration	Parameter
CPU	Intel Core i7-12700
GPU	NVIDIA RTX A5000
Operating system	Windows10
Deep learning framework	Pytorch 1.11.0
Programming language	Python 3.8

**Table 3:** Model performance using different ResNets. A ✔ indicates that transfer learning is used.

Transfer Learning	Method	Val MAE	Val RMSE	Test MAE	Test RMSE	Size (MB)
	ResNet18	25.08	32.68	32.40	42.30	44.7
	ResNet34	27.77	34.14	37.56	46.01	83.3
	ResNet50	24.01	30.95	30.28	41.01	97.8
	ResNet101	24.43	34.56	27.61	39.41	171
✔	ResNet18	17.21	25.15	22.67	34.59	44.7
✔	ResNet34	15.67	19.80	20.79	26.56	83.3
✔	ResNet50	12.15	19.18	17.72	25.15	97.8
✔	ResNet101	12.05	17.75	16.63	25.87	171

**Table 4:** Model performance that fuses different numbers of features.

Number	Val MAE	Val RMSE	Test MAE	Test RMSE
10	12.58	17.90	14.62	21.04
15	10.31	16.20	14.87	23.72
20	10.71	15.45	14.41	22.55
25	10.50	15.81	14.65	23.40
30	11.06	15.25	15.36	23.27

**Table 5:** Model performance with different scale feature convolution kernels.

Scale	Val MAE	Val RMSE	Test MAE	Test RMSE
1.0	10.71	15.45	14.41	22.55
0.5, 1.0, 1.5	10.47	14.98	14.42	20.70
0.6, 1.0, 1.4	10.09	14.61	14.08	20.08
0.7, 1.0, 1.3	9.94	14.91	14.54	22.91
0.8, 1.0, 1.2	9.93	15.01	13.74	22.02
0.9, 1.0, 1.1	10.52	15.57	13.99	20.70
0.5, 0.8, 1.0, 1.2, 1.5	10.19	14.82	13.34	20.57
0.6, 0.8, 1.0, 1.2, 1.4	9.42	15.47	16.45	24.78
0.7, 0.8, 1.0, 1.2, 1.3	9.95	14.98	16.08	24.81
0.8, 0.9, 1.0, 1.1, 1.2	10.11	14.61	12.74	19.40
0.5, 0.8, 0.9, 1.0, 1.1, 1.2, 1.5	9.96	14.85	14.21	22.11
0.6, 0.8, 0.9, 1.0, 1.1, 1.2, 1.4	10.92	15.99	13.72	20.82
0.7, 0.8, 0.9, 1.0, 1.1, 1.2, 1.3	10.29	14.56	13.95	20.39

**Table 6:** Model performance when extracting features from different blocks.

Block	Val MAE	Val RMSE	Test MAE	Test RMSE
3	15.58	22.45	21.13	30.71
4	10.11	14.61	12.74	19.40
3, 4	9.32	12.43	12.39	18.81

**Table 7:** Model performance for different values of λ.

λ	Val MAE	Val RMSE	Test MAE	Test RMSE
0	9.32	12.43	12.39	18.81
$1\times 10^{-7}$	9.43	12.81	12.64	18.46
$1\times 10^{-8}$	9.19	12.61	12.24	18.33
$1\times 10^{-9}$	9.03	12.09	12.07	18.20
$1\times 10^{-10}$	9.29	12.46	12.41	18.77

**Table 8:** The performance of DFCCNet and CSRNet at different density levels.

Method	Density Level	MAE	NAE
CSRNet (baseline)	level1	7.03	0.121
	level2	10.10	0.073
	level3	21.46	0.077
DFCCNet	level1	4.26	0.074
	level2	9.85	0.071
	level3	19.17	0.068

**Table 9:** Experimental results of chicken counting using different methods.

Method	Val MAE	Val RMSE	Test MAE	Test RMSE	Parameters (MB)	FPS
yolov5x	11.69	16.88	15.48	24.92	87.20	36.70
yolov7x	15.61	24.14	18.54	29.06	70.78	41.39
MCNN	12.83	17.71	17.04	22.92	0.13	56.68
CSRNet	10.15	13.78	14.20	21.10	16.26	8.43
CANNet	10.78	14.31	14.17	18.78	18.10	7.90
SGANet	9.77	13.07	14.77	22.62	21.79	5.90
Ours	9.03	12.09	12.07	18.20	17.89	16.15

## Data Availability

The representative dataset can be accessed at https://stuscaueducn-my.sharepoint.com/:u:/g/personal/3170062_stu_scau_edu_cn/ETT-vDigmvZBu6EgSRtSn0sBnNHLojY_tDmiVaoZteVP3g?e=rGa2yO (accessed on 15 December 2022).
